# Free hemoglobin concentration in severe sepsis: methods of measurement and prediction of outcome

**DOI:** 10.1186/cc11425

**Published:** 2012-07-16

**Authors:** Michael Adamzik, Tim Hamburger, Frank Petrat, Jürgen Peters, Herbert de Groot, Matthias Hartmann

**Affiliations:** 1 Klinik für Anästhesiologie und Intensivmedizin, Universitätsklinikum Essen und Universität Duisburg-Essen, Hufelandstr. 55, 45122 Essen, Germany; 2 Institut für Physiologische Chemie, Universitätsklinikum Essen und Universität Duisburg-Essen, Hufelandstr. 55, 45122 Essen, Germany

## Abstract

**Introduction:**

Hemolysis can be induced in sepsis via various mechanisms, its pathophysiological importance has been demonstrated in experimental sepsis. However, no data on free hemoglobin concentrations in human sepsis are available. In the present study we measured free hemoglobin in patients with severe sepsis as well as in postoperative patients using four methods. It was our aim to determine the potential value of free hemoglobin as a biomarker for diagnosis and outcome of severe sepsis in critical illness.

**Methods:**

Plasma concentration of free hemoglobin was determined in patients with severe sepsis (*n *= 161) and postoperative patients (*n *= 136) on day 1 of diagnosis and surgery. For the measurement of free hemoglobin, an enzyme linked immunosorbent assay and three spectrophotometric algorithms were used. Moreover, SAPS II- and SOFA scores as well as procalcitonin concentration and outcome were determined. Kaplan-Meier analysis was performed and odds ratios were determined after classification of free hemoglobin concentrations in a high and low concentration group according to the median. For statistical evaluation the Mann-Whitney test and logistic regression analysis were used.

**Results:**

In non-survivors of severe sepsis, free hemoglobin concentration was twice the concentration compared to survivors. Thirty-day survival of patients, as evidenced by Kaplan-Meier analysis, was markedly lower in patients with high free hemoglobin concentration than in patients with low free hemoglobin concentration. Best discrimination of outcome was achieved with the spectrophotometric method of Harboe (51.3% vs. 86.4% survival, p < 0.001; odds ratio 6.1). Multivariate analysis including free hemoglobin, age, SAPS II- and SOFA-score and procalcitonin demonstrated that free hemoglobin, as determined by all 4 methods, was the best and an independent predictor for death in severe sepsis (p = 0.022 to p < 0.001). Free hemoglobin concentrations were not significantly different in postoperative and septic patients in three of four assays. Thus, free hemoglobin can not be used to diagnose severe sepsis in critical illness.

**Conclusions:**

Free hemoglobin is an important new predictor of survival in severe sepsis.

## Introduction

Sepsis is the third common cause of death in western countries, but despite decades of research its prognosis has not improved markedly [1]. Concerning the pathophysiology leading to sepsis, a generalized inappropriate inflammation has been demonstrated [2]. While localized inflammation is an effective strategy to defend against infection, its generalization in sepsis becomes harmful. Mechanistically, the presence of specific pathogen structures (so-called pathogen-associated molecular patterns) is detected by pattern recognition receptors, including Toll-like receptors, leading to an activation of the immune system and coagulation [3]. Similarly, tissue damage, which can occur during sepsis, leads to the release of endogenous danger-associated molecular patterns, which promote inflammation via Toll-like receptors, too [4].

Free hemoglobin has been demonstrated to affect the inflammatory response in experimental endotoxinemia and sepsis. In 1961, Davis and Yull postulated, that a specific synergism exists between escherichia coli and red blood cells, the combination of which was lethal on intraperitoneal application in animals, whereas neither bacteria nor blood alone were lethal [5]. In 1998, Bloom *et al. *revealed that free hemoglobin increases the lipopolysaccharide (LPS)-induced release of TNFα in rats [6]. These findings were broadened by Su *et al.*, demonstrating increased mortality and TNFα levels in LPS-treated mice after application of hemoglobin [7]. These results were extended by a recent study demonstrating that low grade sepsis in mice induced by cecal ligation and puncture leads to the death in animals deficient for heme oxygenase 1, while the wild type animals survived [8]. Moreover, the authors demonstrate that the mortality in mice is associated with higher free hemoglobin concentrations, that mortality can be decreased by infusion of hemopexin to diminish heme levels and that mortality is increased in patients with low hemopexin concentrations. Another line of evidence comes from the finding in patients with severe sepsis, that a heme oxygenase 1 polymorphism is associated with outcome [9].

The use of biomarkers for the diagnosis and prognosis of severe sepsis is important to early identify patients with the disease, to identify those patients with a bad prognosis and to guide therapy. The capabilities of the currently available sepsis markers, the best of which is procalcitonin, and current clinical and physiological scoring systems, are far from perfect. It is a main problem of sepsis-marker, that a differentiation between critical ill patients (for example, surgical patients) exhibiting limited inflammatory response from septic patients with generalized inappropriate inflammation is difficult. Therefore, we tested whether free hemoglobin determined at day 1 can be used as a biomarker for the diagnosis and prognosis of severe sepsis in critical illness. Determination of free hemoglobin has not been performed in patients with sepsis so far and thus, eventual measuring errors might occur, as no standard method has been established in this setting. Therefore, we determined free hemoglobin with 4 different methods to increase the validity of our results and to find an appropriate method for the measurement in patients with severe sepsis.

In the present study, free hemoglobin concentrations and outcome were determined in patients with severe sepsis to evaluate whether free hemoglobin can be used as a biomarker for outcome in severe sepsis. Moreover, postoperative patients were compared with sepsis patients to evaluate whether free hemoglobin is able to diagnose severe sepsis in patients with severe illness.

## Materials and methods

### Patients

The study was formally and specifically reviewed and approved by the Ethics Committee of the University Hospital Essen, the appropriate institutional review board. Informed written consent was given by probands and postoperative patients. Informed consent of patients with sepsis was waived by the ethics committee, but written informed consent for the use of data was acquired from the surviving patients after recovery from the disease. Over a period of two years, 161 patients admitted to an intensive care unit (ICU) of the University Hospital of Essen were considered eligible for the study if they fulfilled the criteria for severe sepsis (sepsis group) [10]. The second cohort comprised patients admitted to the ICU after surgery, who did not meet the criteria for sepsis (postoperative group, *n *= 136). Two patients in this group died in the postoperative course. Moreover, free hemoglobin concentration was determined in 9 healthy probands. A detailed characterization of patients with sepsis and postoperative controls is given in Table 1. Plasma obtained from EDTA blood, drawn for standard laboratory determinations and centrifuged within 2 hours (15 minutes, 2,500 g), was used for determination of free hemoglobin on day 1 of diagnosis and surgery, respectively. Plasma samples were stored frozen at -70^°^C. Blood was drawn via an indwelling arterial catheter (postoperative and septic patients) or via venous puncture (probands). Procalcitonin concentration, the simplified acute physiology score II (SAPS II), and the sequential organ failure assessment score (SOFA score) were determined on day 1 [11,12]. The amount of packed red blood cell transfusions within 3 days and 4 weeks before surgery or the diagnosis of sepsis, and 30-day survival were also determined.

**Table 1:** Characterization of postoperative patients, survivors and non-survivors of severe sepsis based on 30-day mortality.

	Postoperative patients	Survivors of severe sepsis	Non-survivors of severe sepsis
**Patient characteristics**			
Patients, n	136	111	50
Age, years	60 (46, 69)	60 (48, 72)	56 (49, 66)
Gender, male/female	68/68	64/47	28/22
**Disease severity**			
SAPS II score	21.5 (16.0, 27.3)	44.0 (31.0, 57.0)	54.0 (40.0, 60.5)
SOFA score	3.0 (1.8, 6.0)	11.0 (8.0, 14.0)	12.0 (10.0, 15.0)
Procalcitonin, ng/ml	1.5 (0.5, 3.7)	3.2 (1.0, 16.0)	3.8 (1.1, 16.7)
**Primary diagnosis (*n *=)**			
GI disease	20	31	17
GI cancer	40	16	10
Cancer other	22	7	6
Urogenital disease	6	3	2
Urogenital cancer	18	3	2
Cardiovascular	0	24	4
Lung disease	0	19	5
Lung cancer	0	1	2
Other diseases	30	7	2
**Infection type (in %)**			
Gram positive	0	42.9	34.7
Gram negative	0	36.1	32.6
Fungal	0	14.2	18.4
Cultures negative	0	6.8	14.3
**Kidney function**			
Urea, mg/l	140 (100, 203)	410 (230, 540)	490 (300, 745)
Dialysis, % patients	29.4	45.9	80.0

Included are biometric data, primary diagnosis (which was identical to the type of surgery in the postoperative patients), disease severity, and urea obtained within 24 h of ICU admission. Note, that more than one infection type was determined in several patients. Requirement for dialysis within 30 days is shown. Values are given as median [quartiles] unless stated otherwise. SAPS II score: simplified acute physiology score II; SOFA score: sequential organ failure assessment score.

### Determination of free hemoglobin

Plasma hemoglobin concentration was determined using both ELISA and spectrophotometry. A commercial available ELISA was performed according to the manufacturers' recommendations (Bethyl Laboratories, Montgomery, TX, USA). Three spectrophotometric algorithms described in the literature were used to determine the free hemoglobin concentration from 350 to 750 nm spectra. The algorithms used were described by Harboe, Noe, and Fairbanks (the so-called empirical method) [13-15].

### Determination of biomarkers

For the determination of procalcitonin concentration, the Liaison Brahms PCT assay (Diasorin S.p.A., Sallugia, Italy) was used.

### Statistical analysis

SPSS Version 19 (SPSS Inc., Chicago, Ill, USA) was used for all statistical procedures. An a priori alpha error *P*-value less than 0.05 was considered to indicate statistical significance.

#### Univariate analyses

Free hemoglobin concentrations, as determined by the different methods in patients with and without severe sepsis, are given as median and quartiles. The Shapiro-Wilkes test excluded a normal distribution of the free hemoglobin values obtained with each of the methods used. Therefore, the Mann-Whitney test was used for statistical evaluation and nonparametric correlations and significance levels according to Spearman-Rho were determined to compare the results of the four assays. The median of the hemoglobin concentration of all patients with sepsis was used for the definition of the high and low hemoglobin concentration sub-cohorts. Thereafter, Kaplan-Meier curves were generated to compare the 30-day survival of septic patients with high and low hemoglobin concentration; the log rank test was used for statistical analysis. Odds ratios for death in severe sepsis in the high and low hemoglobin groups were calculated, along with the respective 95% confidence interval (CI) and the significance values using the Chi-square test. Box plots were generated using SPSS to demonstrate the median, quartiles, minimum and maximum values.

#### Multivariate analyses

For multivariate analysis of possible predictors of 30-day mortality, logistic regression was used. Age, SAPS II and SOFA scores, procalcitonin concentration and hemoglobin concentration were included as continuous variables using the backward elimination (likelihood ratio) method using SPSS. Only one of the methods for the determination of free hemoglobin was included in the analysis to avoid multicollinearity. Instead, multivariate analysis was repeated with each of the methods used for the measurement of free hemoglobin. Analyses were also performed with either SAPS II or SOFA score, leading to comparable results, and thus exluding multicollinearity (data not shown).

## Results

### Free hemoglobin in survivors and non-survivors of sepsis

**Figure 1:**
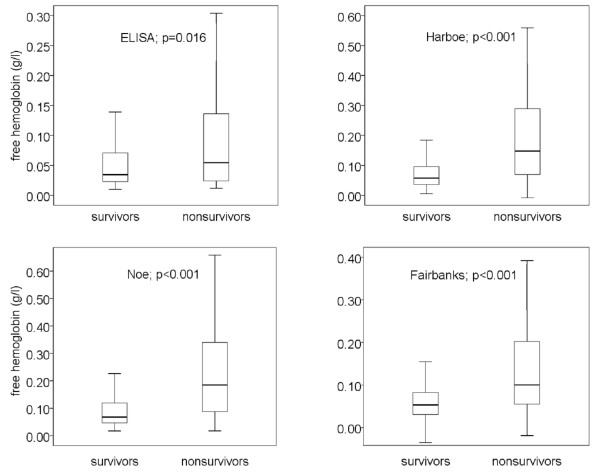
**Free hemoglobin concentrations in survivors and non-survivors of severe sepsis as obtained by ELISA and three spectrophotometric methods**. Data are given in boxplots (median, quartile, minimum, maximum). For statistical evaluation the Mann-Whitney test was used. Spectrophotometric methods were the methods of Harboe [13], Noe [14] and Fairbanks [15].

In the present study 50 out of 161 patients died within 30 days. Comparison of free hemoglobin concentrations in survivors and non-survivors using ELISA and spectrophotometry (three algorithms) demonstrated marked differences: the median of free hemoglobin concentration was increased by about 100% in non-survivors, as evidenced by all four methods used (*P *= 0.016 to < 0.001, Figure 1). Medians (quartiles) in survivors and non-survivors obtained with the four methods were as follows: ELISA 0.034 g/l (0.023, 0.071) vs. 0.054 g/l (0.024, 0.1389), Harboe 0.057 g/l (0.036, 0.098) vs. 0.147 g/l (0.069, 0.290), Noe 0.068 g/l (0.047, 0.123) vs. 0.185 g/l (0.087, 0.344) and Fairbanks 0.053 g/l (0.030, 0.085) vs. 0.100 g/l (0.055, 0.208).

**Figure 2:**
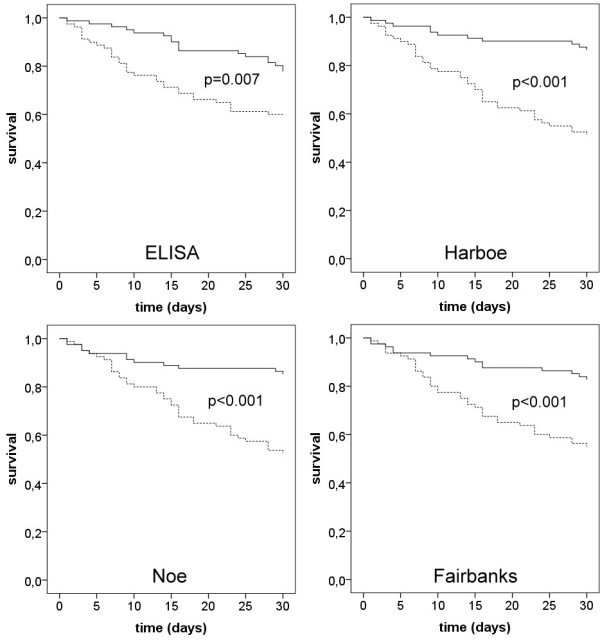
**Kaplan-Meier analyses of 30-day survival of patients with severe sepsis demonstrating the effect of high and low free hemoglobin concentrations as determined by four methods**. Four sets of Kaplan-Meier curves are shown, as analysis was performed with each of the four methods used for the determination of free hemoglobin. The continuous lines show the survival of patients with free hemoglobin concentrations lower than the median, and the dotted lines show the survival when free hemoglobin concentration was higher than the median of all patients with sepsis. Significance levels for the log rank test are given in the figure. Methods used to measure free hemoglobin were ELISA and the spectrophotometric methods of Harboe [13], Noe [14] and Fairbanks [15].

In order to generate Kaplan-Meier curves, patients with severe sepsis were divided in two sub-cohorts according to the median of the free hemoglobin concentrations as determined by the four methods. Higher concentrations of free hemoglobin on the first day of the diagnosis of severe sepsis were associated with significantly increased 30-day mortality (Figure 2). Remarkably, this association could be demonstrated for each of the four methods used. Best discrimination between survivors and non-survivors was obtained using the method of Harboe (86.4% vs. 51.3% survival, *P *< 0.001). The odds ratios for septic patients to die within 30-days, when free hemoglobin concentration exceeded the median, were 2.3 (ELISA), 6.1 (Harboe), 5.2 (Noe), and 3.9 (Fairbanks) (Table 2).

**Table 2:** Univariate analyses demonstrating the association of (A) free hemoglobin concentration and (B) age, SAPS II score, SOFA score, and procalcitonin with 30-day survival in severe sepsis.

**A**.	Median	Odds ratio	95% CI	Significance level
**ELISA**	0.037 g/l	2.33	1.17, 4.65	0.015
**Harboe**	0.068 g/l	6.06	2.79, 7.80	< 0.001
**Noe**	0.085 g/l	5.21	2.44, 11.11	< 0.001
**Fairbanks**	0.059 g/l	3.92	1.90, 8.06	< 0.001

**B**.	**Median**	**Odds ratio**	**95% CI**	**Significance level**

**Age**	57.0 y	1.22	0.63, 2.38	0.560
**SAPS II score**	47.5	1.92	0.98, 3.79	0.058
**SOFA score**	11.5	2.07	0.97, 4.43	0.058
**Procalcitonin**	3.385 ng/ml	1.42	0.68, 2.95	0.351

Patients were grouped according to high and low free hemoglobin determined by the median of the respective value, measured by ELISA, and by the Harboe, Noe and Fairbanks methods [13-15]. Thereafter, odds ratios for death in severe sepsis were determined. Depicted are the median of the respective value, the odds ratios, the 95% confidence intervals (95% CI), and the significance levels (Chi square test). SAPS II score: simplified acute physiology score II; SOFA score: sequential organ failure assessment score.

### Comparison of free hemoglobin concentration, SAPS II and SOFA score, and procalcitonin concentration as predictors of survival in sepsis

To compare the importance of free hemoglobin concentration as a predictor of survival in severe sepsis with other established markers and scores, both univariate analyses and multivariate analyses were performed. Univariate analyses demonstrated that free hemoglobin as determined with any of the four methods was a good predictor of death in severe sepsis, while SAPS II score, SOFA score, and procalcitonin concentration were not statistically significant in this study (Table 2).

In the multivariate analyses we included age, SAPS II and SOFA scores, procalcitonin concentration as well as free hemoglobin values obtained by one of the methods. The analysis was repeated for each method of free hemoglobin determination to avoid multicollinearity. The results confirm that free hemoglobin concentration, irrespective of the method used, is the best and an independent predictor of survival in severe sepsis in this study. Significance levels for the four methods were as follows: *P *= 0.022 for ELISA, *P *< 0.001 for Harboe, *P *< 0.001 for Noe, and *P *< 0.002 for the Fairbanks method. To exclude collinearity between SAPS II and SOFA scores, analysis was repeated with either the SAPS II score or the SOFA score. The results were almost identical and thus exclude multicollinearity as the reason for the results of the multivariate analysis (data not shown).

### Free hemoglobin in postoperative patients, patients with severe sepsis and healthy probands

**Figure 3:**
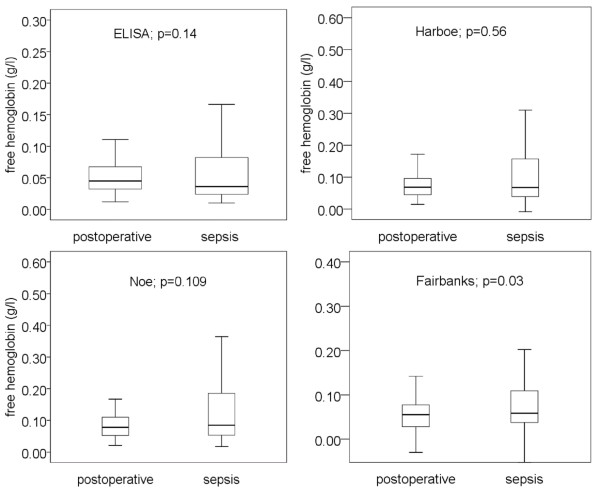
**Free hemoglobin measured in postoperative patients and patients with severe sepsis using four methods**. Free hemoglobin did not differ in postoperative and septic patients using three of the four methods. Therefore, free hemoglobin cannot serve as a biomarker for the diagnosis of severe sepsis in critical illness. Data are given in boxplots (median, quartile, minimum, maximum). For statistical evaluation the Mann-Whitney test was used. Methods used to measure free hemoglobin were ELISA and the spectrophotometric methods of Harboe [13], Noe [14] and Fairbanks [15].

In postoperative patients (*n *= 136), median free hemoglobin concentrations, as determined by ELISA and three different spectrophotometric methods, were in the range between 0.045 and 0.078 g/l (Figure 3). The median values for free hemoglobin concentrations in septic patients (= 161), as determined by these methods, were in the range between 0.037 and 0.085 g/l. With the exception of the Fairbanks method, no method showed significant differences in free hemoglobin concentrations between postoperative and septic patients (Figure 3). Thus, free hemoglobin cannot be used to diagnose severe sepsis in critical illness.

Determination of free hemoglobin in nine healthy probands did not demonstrate a significant difference compared to postoperative patients and patients with severe sepsis. Median concentrations (quartiles), as determined by the methods of Harboe, Noe, and Fairbanks were 0.094 g/l (0.078, 0.143), 0.109 g/l (0.087, 0.153) and 0.063 g/l (0.023, 0.121), respectively.

### Correlation of the four methods used for the determination of free hemoglobin

To compare the agreement of the four methods used for determination of free hemoglobin concentration, we determined the nonparametric correlation coefficient and significance level according to Spearman-Rho in postoperative and septic patients. Correlation between the spectrophotometric methods was best for the methods of Noe and Harboe with a correlation coefficient > 0.9 in both postoperative and septic patients. The Spearman-Rho test demonstrated that correlation between all methods used for the determination of free hemoglobin was highly significant (*P *< 0.001). Data are summarized in Table 3.

**Table 3:** Nonparametric correlation coefficients between the free hemoglobin concentrations obtained with four different methods.

Assay method	ELISA	**Harboe **[13]	**Noe **[14]	**Fairbanks **[15]
**ELISA**	1	0.407	0.412	0.408
	1^a^	0.534^a^	0.510^a^	0.484^a^
	1^b^	0.552^b^	0.528^b^	0.512^b^

**Harboe**	0.407	1	0.937	0.552
	0.534^a^	1^a^	0.988^a^	0.806^a^
	0.552^b^	1^b^	0.989^b^	0.774^b^

**Noe**	0.412	0.937	1	0.557
	0.510^a^	0.988^a^	1^a^	0.791^a^
	0.528^b^	0.989^b^	1^b^	0.771^b^

**Fairbanks**	0.408	0.552	0.557	1
	0.484^a^	0.806^a^	0.791^a^	1^a^
	0.512^b^	0.774^b^	0.771^b^	1^b^

Nonparametric correlation coefficients for postoperative patients are given without superscript letters; ^a^coefficients for septic patients; ^b^coefficients obtained from all patients. Significance levels according to Spearman-Rho were lower than 0.001 for all correlations.

### Influence of red blood cell transfusion on free hemoglobin concentration

Irrespective of the method used to determine free hemoglobin concentration, there was only one significant positive correlation between the free hemoglobin concentration and the number of packed red blood cell units transfused within either 3 days or 4 weeks before surgery or the diagnosis of severe sepsis, respectively. This significant correlation (between the Noe method and transfusion within 4 weeks) was not confirmed by the other methods and thus might best be explained by the fact that we did not correct for repeat measurements. The details of analysis of free hemoglobin and transfusions are given in Table 4. Moreover, the number of packed red blood cell units transfused to patients before the onset of sepsis was not different in non-survivors of sepsis, with a median (quartiles) of 0.0 (0.0, 0.0) at 3 days and 0.0 (0.0, 3.0) at 4 weeks, compared to survivors of sepsis in whom the median (quartiles) was 0.0 (0.0, 0.0) at 3 days and 0.0 (0.0, 4.0) at 4 weeks (*P *= 0.78 and 0.54, respectively.

**Table 4:** Lack of significant positive correlation between free hemoglobin concentration, as determined with four methods and packed red blood cell units transfused within 3 days or 4 weeks before surgery, and diagnosis of severe sepsis, respectively.

	ELISA	**Harboe **[13]	**Noe **[14]	**Fairbanks **[15]
	3 days/4 weeks	3 days/4 weeks	3 days/4 weeks	3 days/4 weeks
**Postoperative patients**				
Correlation coefficient	0.066/0.076	0.168/0.158	0.169/0.178	0.084/0.094
Significance level	0.455/0.381	0.510/0.066	0.050/0.039	0.331/0.278
**Patients with sepsis**				
Correlation coefficient	-0.154/-0.093	-0.204/-0.039	-0.204/-0.041	-0.217/-0.088
Significance level	0.51/0.244	0.01/0.623	0.009/0.604	0.006/0.271

Given are nonparametric correlation coefficients and significance levels according to Spearmen-Rho.

## Discussion

The present study demonstrates that free hemoglobin concentrations above the median value, measured in plasma obtained from patients with severe sepsis on the first day of the diagnosis, are associated with increased 30-day mortality. Free hemoglobin concentration proved to be a better marker of outcome than procalcitonin, SAPS II or SOFA scores.

### Measurement of free hemoglobin

Measurement of proteins in plasma samples may be affected by differences in the composition of proteins, lipids, and small molecules, which may occur when comparing samples from postoperative patients and patients with sepsis. For this reason, we used four methods for the determination of free hemoglobin, an ELISA, and spectrophotometry using three algorithms. Of the three spectrophotometric methods, one algorithm corrects for bilirubin, while the other two methods do not. Despite these differences in methodology, all spectrophotometric methods showed a marked, approximately two-fold increase in free hemoglobin concentration in non-survivors compared to survivors. Moreover, the results are confirmed by those obtained from a commercially available hemoglobin ELISA. The results of all methods are comparable: correlation between the methods was high, all methods showed an approximately two-fold increase in free hemoglobin concentration and mean values in healthy probands, postoperative and septic patients were similar using the four methods. Thus, there is ample evidence that the results reflect true changes in free hemoglobin concentrations.

While there is clear evidence for the detrimental effects of free hemoglobin in animal models of endotoxinemia and sepsis (see next paragraph), our study is the first to report free hemoglobin measurements in humans with severe sepsis. Interestingly, our results also demonstrate that the free hemoglobin concentrations show no significant differences between healthy probands, postoperative patients, and patients with severe sepsis (survivors and non-survivors combined) on the first day of surgery and diagnosis, respectively. Thus, free hemoglobin cannot serve as a biomarker for the diagnosis of severe sepsis. Importantly however, free hemoglobin measured at day 1 was markedly higher in non-survivors of severe sepsis than in survivors. If the free hemoglobin concentrations were above the median in patients with severe sepsis, the prognosis was poor. Odds ratios for death within 30 days in patients with severe sepsis were between 2.3 and 6.1 depending on the method used for the determination of free hemoglobin concentration, thus demonstrating a major effect on mortality. Moreover, both univariate and multivariate analyses demonstrate that free hemoglobin, as determined with four different methods is a far better predictor of death in sepsis than age, SAPS II score, SOFA score, and procalcitonin concentration. The finding that free hemoglobin concentration as determined in all patients with severe sepsis, was not significantly elevated in comparison to postoperative patients, can be explained by our finding that an increase occurred only in those patients with a poor prognosis. In summary, the present study demonstrates an important link between free hemoglobin concentration in plasma and the outcome of severe sepsis.

### Potential mechanisms leading to increased free hemoglobin in sepsis

In principle, increased free hemoglobin concentrations in patients with a poor prognosis might be due to an increased hemolysis and/or a reduced degradation by endogenous scavenger and detoxification pathways, such as hemoglobin stabilizing protein, haptoglobin, hemopexin, heme oxygenase-1, and CD 163 [16]. Concerning potential mechanisms leading to hemolysis in sepsis, various mechanisms have been proposed. First, some pathogens are capable of inducing hemolysis via toxins and it has been convincingly demonstrated that hemolytic activity is an important virulence factor [17-19]. Second, fibrin strands generated by septic disseminated intravascular coagulation can destroy erythrocytes leading to so called schistocytes [20,21]. Third, the complement system, which is activated during sepsis, may impair the viability of erythrocytes [22]. Fourth, lipopolysaccharides can affect mechanical membrane properties, thus potentially promoting cell destruction [23]. Finally, sepsis has been claimed to induce death of erythrocytes by eryptosis, a mechanism that shares similarities with apoptosis [24].

Further, increased free hemoglobin concentrations might be due to transfusion of packed red blood cells. However, in our study no association of free hemoglobin concentration with transfused red blood cell units was detectable in either postoperative or septic patients.

### Pathophysiological importance of hemolysis

There are different lines of evidence showing a potential pathophysiological importance of hemolysis. The harmful effects of blood transfusion, called storage lesions, are thought to be caused by hemolysis [25]. Infusion of free hemoglobin to mice treated with LPS increased TNFα concentration and mortality, and these effects were blocked by hemoglobin antibodies [6,26]. Generation of free hemoglobin complexes with LPS result in enhanced biological activity, and free hemoglobin and heme act synergistically at various Toll-like receptors [27-29]. Moreover, reduced heme degradation in heme oxygenase-1-deficient mice with experimental sepsis is associated with increased mortality in comparison to wild type mice, and increased free hemoglobin concentrations in mice with high grade cecal ligature and puncture in comparison to a low grade lesion has been demonstrated [8]. In line with this finding are the results of a recent study demonstrating that genotypes of a heme oxygenase 1 polymorphism affect the outcome of patients with adult respiratory distress syndrome and that decreased hemopexin plasma concentrations are indicative of poor prognosis in sepsis [8,9]. Another aspect of the pathophysiological importance of free hemoglobin is the effective nitric oxide scavenging property of the molecule, causing microvascular perfusion disturbances and an increase in arterial blood pressure [30].

Further mechanisms, apart from those based on free hemoglobin, might be involved in the deleterious effects of hemolysis in sepsis. Ferrous iron has been demonstrated to catalyze the generation of radicals, which can modify lipids, proteins and DNA, thus inducing inflammation [31]. Moreover, the finding that the growth of microbes is critically dependent on iron might serve as a further explanation for the detrimental effects of hemolysis [32,33]. Hemolysis may also result in the generation of microparticles, which are able to induce inflammation and disseminated intravasular coagulation [34].

### Is there a causal relationship of free hemoglobin concentration and outcome in severe sepsis?

While the results of the present study clearly demonstrate a strong association of free hemoglobin with the outcome of patients with sepsis, it is not possible in general to prove a causal relationship in an observation study. As stated above, however, there is ample evidence for a causal relationship between free hemoglobin and outcome of sepsis in animal models: free hemoglobin affects Toll-like receptor signal transduction, TNFα generation and mortality [6,29,35]. In addition, the effects of free hemoglobin in experimental sepsis can be inhibited by hemopexin [8]. Moreover, recent studies demonstrate the mechanisms leading to the unfavorable effects of free hemoglobin including oxidation and production of ferryl hemoglobin and heme generation [36-38]. In humans, the genotypes of a heme oxygenase-1 polymorphism in adult respiratory distress syndrome are associated with outcome [9]. Taken together, these findings suggest an outstanding importance of hemoglobin and its derivatives. The results of our study are in accordance to these experimental findings: however, an association of free hemoglobin with outcome is no proof for causality. Moreover, it seems necessary to investigate the eventual contribution of free iron and microparticles to the deleterious effects of hemolysis in human severe sepsis.

### Which method for free hemoglobin measurement might be best suitable as predictor of death in severe sepsis?

The results demonstrate that all methods used are appropriate as predictors of death in severe sepsis. However, odds ratios were higher with spectrophotometric assays than with the ELISA. Although we can only speculate on the reasons for this result, it is advantageous that the best prediction of death was achieved with the very simple and inexpensive spectrophotometric method of Harboe. However, further studies are necessary to determine the best method for determination of free hemoglobin in severe sepsis.

## Conclusions

The results of the present study reveal that concentrations of free hemoglobin above the median value measured on the first day of severe sepsis are associated with increased 30-day mortality. The results suggest that the experimental findings of detrimental effects of free hemoglobin in experimental models may be relevant in patients with severe sepsis.

## Key messages

• Free hemoglobin is a better predictor of outcome than procalcitonin, SAPS II and SOFA scores.

• The increase of free hemoglobin concentration on day 1 of severe sepsis in patients with a poor prognosis is compatible with the notion from experimental findings of an involvement of free hemoglobin in the pathophysiology of severe sepsis.

• The spectrophotometric method of Harboe is a simple and inexpensive method for the prediction of outcome in severe sepsis.

## Abbreviations

CI: confidence interval; ELISA: enzyme linked immunosorbent assay; LPS: lipopolysaccharide; OR: odds ratio; SAPS II score: simplified acute physiology score II; SOFA score: sequential organ failure assessment score; TNFα: tumor necrosis factor alpha.

## Competing interests

The authors declare that they have no competing interests.

## Authors' contributions

Conception of the study was by TH, FP, HdG, MH. Patient data acquisition was performed by MA. TH and FP performed measurements of free hemoglobin. Analysis of data and drafting of the manuscript was performed by MH. MA, TH, FP, HdG, JP, and MH critically revised and approved the manuscript. All authors have read and approved the manuscript for publication.
